# MicroRNA-Dependent Regulation of Transcription in Non-Small Cell Lung Cancer

**DOI:** 10.1371/journal.pone.0090524

**Published:** 2014-03-13

**Authors:** Sonia Molina-Pinelo, Gabriel Gutiérrez, Maria Dolores Pastor, Marta Hergueta, Gema Moreno-Bueno, Rocío García-Carbonero, Ana Nogal, Rocío Suárez, Ana Salinas, Francisco Pozo-Rodríguez, Fernando Lopez-Rios, Maria Teresa Agulló-Ortuño, Irene Ferrer, Asunción Perpiñá, José Palacios, Amancio Carnero, Luis Paz-Ares

**Affiliations:** 1 Molecular Oncology and New Therapies Group. Instituto de Biomedicina de Sevilla (IBIS) (HUVR, CSIC, Universidad de Sevilla), Sevilla, Spain; 2 Genetic Department, Universidad de Sevilla, Seville, Spain; 3 Biochemistry Department, Universidad Autónoma de Madrid, Instituto de Investigaciones Biomédicas ‘Alberto Sols’ CSIC-UAM, Madrid, Spain; 4 Medical Oncology Department, Hospital Universitario Virgen del Rocio, Sevilla, Spain; 5 Instituto de Ciências Biomédicas Abel Salazar (ICBAS), Porto, Portugal; 6 Service of Neumology, Hospital 12 de Octubre, Madrid, Spain; 7 Centro de Investigación Biomédica en Red de Enfermedades Respiratorias (CIBERES), Madrid, Spain; 8 Pathology Department, Laboratorio de Dianas Terapéuticas, Centro Integral Oncológico Clara Campal, Madrid, Spain; 9 Service of Oncology, Instituto de Investigación Sanitaria i+12, Hospital Doce de Octubre, Madrid, Spain; 10 Service of Neumology, Hospital Severo Ochoa, Madrid, Spain; 11 Department of Pathology, Hospital Universitario Ramón y Cajal, Instituto de Investigación Sanitaria Ramón y Cajal (IRYCIS), Madrid, Spain; 12 Molecular Biology of Cancer Group, Instituto de Biomedicina de Sevilla (IBIS)/(HUVR, CSIC, Universidad de Sevilla), Seville, Spain; 13 IdiPAZ (Instituto de Investigación Sanitaria La Paz) & Fundación MD Anderson International, Madrid Spain; IPMC, CNRS UMR 7275 UNS, France

## Abstract

Squamous cell lung cancer (SCC) and adenocarcinoma are the *most common* histological subtypes of non-small cell lung cancer (NSCLC), and have been traditionally managed in the clinic as a single entity. Increasing evidence, however, illustrates the biological diversity of these two histological subgroups of lung cancer, and supports the need to improve our understanding of the molecular basis beyond the different phenotypes if we aim to develop more specific and individualized targeted therapy. The purpose of this study was to identify microRNA (miRNA)-dependent transcriptional regulation differences between SCC and adenocarcinoma histological lung cancer subtypes. In this work, paired miRNA (667 miRNAs by TaqMan Low Density Arrays (TLDA)) and mRNA profiling (Whole Genome 44 K array G112A, Agilent) was performed in tumor samples of 44 NSCLC patients. Nine miRNAs and 56 mRNAs were found to be differentially expressed in SCC versus adenocarcinoma samples. Eleven of these 56 mRNA were predicted as targets of the miRNAs identified to be differently expressed in these two histological conditions. Of them, 6 miRNAs (miR-149, miR-205, miR-375, miR-378, miR-422a and miR-708) and 9 target genes (CEACAM6, CGN, CLDN3, ABCC3, MLPH, ACSL5, TMEM45B, MUC1) were validated by quantitative PCR in an independent cohort of 41 lung cancer patients. Furthermore, the inverse correlation between mRNAs and microRNAs expression was also validated. These results suggest miRNA-dependent transcriptional regulation differences play an important role in determining key hallmarks of NSCLC, and may provide new biomarkers for personalized treatment strategies.

## Introduction

Lung cancer is the primary cause of cancer death worldwide, being responsible for one million deaths annually [1]. Non-small cell lung cancer (NSCLC) accounts for 80% of all lung tumours, and includes several histological subtypes such as large cell carcinoma (LCC), squamous cell carcinoma (SCC), and adenocarcinoma. SCC and adenocarcinoma are the *most common* types of NSCLC, accounting for 25% and 40% of all cases, respectively [2], [3]. SCC derives from dysplastic multilayer epithelium in the *central* airways, whereas adenocarcinoma originates preferentially from precursor cells of the mono- or bilayer surface epithelium of the lung periphery [4].

*NSCLC, regardless of the histological subtype, has* been traditionally treated in the clinic as a single homogeneous entity. However, increasing evidence illustrates the great biological diversity of this disease, which is progressively leading to more specific diagnostic and therapeutic strategies depending upon the histological subtype concerned. Indeed, advances in targeted lung cancer therapy now demand accurate classification of NSCLC [5]. For example, EGFR mutations are more prevalent in patients with lung adenocarcinoma, and the presence of these mutations is associated with sensitivity to EGFR tyrosine kinase inhibitors [6]. Similarly, ALK traslocations, present in only 4% of adenocarcinomas, are predictive of a high sensitivity to ALK-directed therapies such as crizotinib. By contrast, FGFR1 amplification is more commonly observed in SCC, and is now being considered a potentially actionable target in clinical trials with FGFR inhibitors [7]. Therefore, a greater knowledge of the molecular mechanisms involved in the genesis, progression and spread of the different subtypes of NSCLC is necessary for the development of specific diagnostic methods and the design of more adequate, individualized and effective therapeutic strategies.

The great advances in genomic technologies have generated many candidate biomarkers with potential clinical value in NSCLC. MicroRNAs, as post-transcriptional modulators, are key players in the regulation of many biological processes. Dysregulation of their physiological roles contributes to many pathological conditions, including the initiation and progression of cancer. In this context, a number of studies have assessed the potential role of miRNA signatures to discriminate histological subtypes or to predict recurrence or survival of NSCLC patients [8], [9], [10], [11], [12], [13], [14], and miRNA profiling has been proposed as a highly reliable strategy for classifying NSCLCs [11], [15], [16]. Nevertheless, the high complexity of transcriptome regulation complicates the full understanding of gene regulatory networks involved in these processes.

To address this issue, the aim of this study was to assess miRNA-dependent transcriptional regulation differences between SCC and adenocarcinoma histological lung cancer subtypes. With this purpose, miRNA and mRNA paired expression profiles were analyzed in NSCLC tumor samples, and the potential interactions among them were explored. In this study we have identified and validated a subset of deregulated miRNAs and target genes that are able to define distinct molecular features of these two major histological subtypes of NSCLC.

## Materials and Methods

### Patients and Tumor Specimens

Patients included in this study were required to have histologically confirmed early stage SCC or adenocarcinoma NSCLC. Tumor samples from 85 patients were prospectively collected during the surgical procedure and immediately snap-frozen at −80°C until further use. Adjacent non-tumor lung tissue was also collected from patients included in the validation cohort. The study protocol was approved by the institutional review boards of participating centers [Hospital Universitario Doce de Octubre (Madrid) and Hospital Universitario Virgen del Rocío (Sevilla)] and all patients provided written informed consent prior to study entry. Clinical and pathologic data were extracted from the medical records and centrally reviewed for the purpose of this study. The study population was divided in a training cohort (N = 44) that was used for profile development and an independent validation cohort (N = 41). Main characteristics of study population are summarized in Tables 1 and 2.

**Table 1:** Summary of patients’ characteristics of the training cohort.

Variable	Subvariable	Squamous cell carcinoma (N = 25)	Adenocarcinoma (N = 19)
***Age***	*years*	71 [62–74]	65 [59–74]
***Gender***	Male	25 (100%)	17 (89.4%)
	Female	–	2 (10.6%)
***Stage***	I–IIB	17 (68%)	16 (84.2%)
	IIIA	7 (28%)	2 (10.5%)
	IIIB–IV	1 (4%)	1 (5.3%)
***Smoking Status***	Current smoker	9 (36%)	7 (36.8%)
	Ex-smoker	16 (64%)	10 (52.7%)
	Never smoker	–	2 (10.5%)

Continuous variables are expressed as median [interquartile range (IQR)] and categorical ones as number of cases (%).

**Table 2:** Summary of patients’ characteristics of the validation cohort.

Variable	Subvariable	Squamous cell carcinoma (N = 25)	Adenocarcinoma (N = 19)
***Age***	*years*	66 [60–74]	66 [63–73]
***Gender***	Male	15 (88.2%)	16 (66.7%)
	Female	2 (11.8%)	8 (33.3%)
***Stage***	I–IIB	15 (88.2%)	21 (87.5%)
	IIIA	2 (11.7%)	2 (8.3%)
	IIIB–IV	–	1 (4.2%)
***Smoking Status***	Current smoker	9 (52.9%)	6 (25.0%)
	Ex-smoker	8 (47.1%)	15 (62.5%)
	Never smoker	–	3 (12.5%)

Continuous variables are expressed as median [interquartile range (IQR)] and categorical ones as number of cases (%).

### Microarray Gene Expression Profiles

Microarray experiments were performed using Human Whole Genome 44 K array G4112A (Agilent technologies, Wilmington, DE). RNA was isolated using Trizol (Invitrogen) and RNAesy Extraction Kit (QIAGen, Germany) as indicated by the manufacturers. RNA was labeled and array hybridized using the Low RNA Linear Amplification Kit and the In Situ Hybridization Kit Plus (Agilent technologies, Wilmington, DE) respectively. After hybridization and washing, the slides were scanned in an Axon GenePix Scanner (Axon Instruments Inc., Union City, CA) and analyzed using Feature Extraction Software 6.1.1 (Agilent technologies, Wilmington, DE). RNA from tumor samples were labeled with Cy5-dUTP, and hybridized against a lung cancer reference pool (labeled with with Cy3-dUTP) consisting of primary tumor tissue from patients with different histological subtypes of lung cancer. As control, ten additional hybridizations were performed using the reciprocal fluorochrome labeling.

To detect differentially expressed genes between the two histological subtypes, two types of analysis were undertaken with the MIDAW tool [17]. First, a t-test was performed with false discovery rate (FDR) control estimated using the single-step Bonferroni procedure. Genes that passed the t-test filter were subjected to a second filter. Only genes showing a mean log ratio value lower that −0.3 or greater than 0.3 (equivalent to a 2-fold change) were selected as differentially expressed. Second, a discriminant analysis for the identification of the set of best marker genes was performed based on the Prediction Analysis of Microarray (PAM) algorithm. Microarray raw data tables have been deposited in the Gene Expression Omnibus under the accession number of GSE42998.

### MicroRNA qRT-PCR Assay

Total RNA, containing small RNA, was extracted from tumour tissue samples by mirVana miRNA isolation kit (Ambion, Austin, TX, USA) according to the manufacturer’s instructions. The total RNA yield was determined using a Nanodrop ND-1000 spectrophotometer (Nanodrop Tech, DE, USA). The Agilent 2100 Bioanalyzer was used to determine the quantity and quality of the RNA samples (Agilent, Palo Alto, CA). Mature human miRNA expression was detected and quantified using the TaqMan Low Density Arrays (TLDA) based on Applied Biosystems’ 7900 HT Micro Fluidic Cards (Applied Biosystems, CA, USA) according to the manufacturer’s instructions. The Human MicroRNA Card Set v2.0 array (Catalog Number 4400238) is a two card set containing a total of 384 TaqMan MicroRNA Assays per card to enable accurate quantification of 667 human microRNAs, all catalogued in the miRBase database. TLDAs were performed in a two-step process. Briefly, during the first step, 450 ng of total RNA were reverse transcribed using Megaplex RT Primers and the TaqMan miRNA reverse transcription kit in a total volume of 7.5 µl. The 7.5 µl reactions were incubated in a G-Storm Thermal Cycler (Gene Technologies, Essex, UK) for 2 min at 16°C, 1 min at 42°C, and 1 min at 50°C during 40 cycles, held for 5 min at 85°C, and then kept at 4°C. In the second step, 6 µL of cDNA sample and TaqMan Universal PCR master mix were loaded in fill ports on the TLDA microfluidic card. The card was briefly centrifuged for 1 min at 331 g to distribute samples in the multiple wells connected to the fill ports and then sealed to prevent well-to-well contamination. The reactions were incubated in a 384 well plate at 50°C for 2 sec and 94.5°C for 10 min, followed by 40 cycles of 30 sec at 97°C and 1 min at 59°C. Finally, the cards were processed and analyzed on an ABIPrism 7900 HT Sequence Detection System. TLDA raw data tables have been deposited in the Gene Expression Omnibus under the accession number of GSE43000. Expression of target miRNAs was normalized to the expression of RNU48. One non-human miRNA, was used in each experiment as a negative control. Cycle threshold (Ct) values were calculated using the SDS software v.2.3 using automatic baseline settings and a threshold of 0.2. Relative quantitation of miRNA expression was calculated by the 2^−ΔCt^ method (Applied Biosystems user bulletin no. 2 (P/N 4303859)). Only miRNA detectable in at least 80% of samples were considered for evaluation. Significance of miRNA expression differences observed between the two histolofical subgroups (adenocarcinoma and SCC) was assessed by the t-test.

### mRNA and miRNA Expression Correlation Assessment

To evaluate the potential association between differentially expressed mRNA and miRNA observed in our study, we searched for the transcriptional targets of the identified miRNAs in three web databases for miRNA target prediction: miRanda [18], TargetScan release 6.0 [19], and miRWalk [20]. Putative target genes that matched with those found to be disregulated in our patient population were selected for further validation by qPCR.

### Validation of Microarray Gene Expression Profiles by qPCR

Eleven differentially expressed genes among the two study conditions (SCC and adenocarcinoma NSCLC), identified as putative targets of several dis-regulated miRNAs, were selected for further validation by qPCR in the original training cohort and then in an independent validation cohort. The RNA was reverse transcribed to cDNA with the High Capacity cDNA Reverse Transcription Kit (Applied Biosystems). Briefly, single-stranded cDNA was synthesized from 1 µg total RNA in 10 µL reaction volume, according to the manufacturer’s protocol. The reaction was incubated at 25°C for 10 min followed by 120 min at 37°C and inactivation at 85°C for 5 min. The TaqMan Gene Expression Assay system (Applied Biosystems) was used for quantitating transcription levels of selected genes (*CEACAM6, CGN, CLDN3, ABCC3, MLPH, ACSL5, TMEM45B, MUC1, DMRT2, DSC3* and *KRT6A).* Three endogenous control genes (*B2M*, *ACTB* and *GAPDH*) and one no-template-control (NTC) were also run for each RNA sample. We chose *B2M* for normalization across different genes as this gene showed the most relatively constant expression across different tissue samples (data not shown). The gene expression for each gene was determined using the median expression level of the three technical replicates. PCR reactions were performed on an Applied Biosystems 7900HT Sequence Detection system in 10 µL volumes at 95°C for 10 min, followed by 40 cycles of 95°C for 15 sec and 60°C for 1 min. Ct values were obtained with the SDS software v.2.3 (Applied Biosystems). Relative quantification of mRNA expression was calculated by the 2^−ΔCt^ method (Applied Biosystems user bulletin no. 2 (P/N 4303859)).

### Validation of miRNA TLDA Expression Profiles by qPCR

Expression of nine selected miRNAs (miR-149, miR-205, miR-375, miR-378, miR-422a, miR-483-5p, miR-494, miR-601 and miR-708) was assessed in the independent validation cohort by the specific TaqMan MicroRNA assays according to the manufacturer’s instructions (Applied Biosystems). Briefly, 2 ng/µL of total RNA was converted into cDNA by reverse transcriptase reaction that was performed by sequential incubation at 16°C for 30 min, 42°C for 30 min and 85°C for 5 min. PCR reaction mixture (10 µL) contained 0.66 µL of RT product, 5 µL of TaqMan 2X Universal PCR Master Mix and 0.5 µL of the appropriate TaqMan MicroRNA Assay (20X) containing primers and probe for the miRNA of interest (Applied Biosystems). The mixture was initially incubated at 95°C for 10 min, followed by 40 cycles of 95°C for 15 seconds and 60°C for 60 seconds. MicroRNA expression was quantified by the comparative 2^−ΔΔCt^ method, normalizing Ct values to RNU48. In the validation cohort, tumor expression values were additionally normalized to expression values in paired adjacent normal lung tissue.

### 3′-UTR Reporter Assay for miR Target Validation

Confirmation of miR-149-binding to the 3′ UTR of ABCC3 and of miR-378 and miR-422-binding to the 3′ UTR of TMEM45B. HEK 293 cells at 80% confluency were co-transfected with luciferase reporter plasmids harboring the complete 3′-UTR of the desired gene (SwitchGear Genomics) along with 100 nM of each miR-mimic or miRNA control (Sigma). DharmaFECT Duo (Thermo Scientific) was used as the transfection reagent in *Opti-MEM* (Life Technologies). Luminescence was assayed 24 hours later using LightSwitch Assay Reagents (SwitchGear Genomics) according to the manufacturer’s instructions. Knockdown was assessed by calculating luciferase signal ratios for specific miRNA/non-targeting control, using empty reporter vector as control for non-specific effects. Each experiment was performed in triplicate. t -test was performed for wells from multiple experiments, and we compared mimic-transfected cells with a mimic control for each gene vector.

### Diagnostic Performance Assessment of Selected Genes

Diagnostic performance parameters were calculated for selected genes in 2x2-contingency tables. Confidence intervals for these parameters were calculated with the Pearson method based on the F distribution. As sensitivity, specificity, Positive Predictive Value (PPV) and Negative Predictive Value (NPV) are statistical measures of the performance of a binary classification test, gene expression values were converted to binary variables with the median expression value as the reference value (high versus low expression). These parameters were calculated for each validated miRNA/target mRNA pair. The concurrent occurence of high miRNA expression and low target mRNA expression in the appropriate histological condition (SCC or adenocarcinoma) was considered a true positive test. Sensitivity or true positive rate measures the proportion of actual positives which are correctly identified. Specificity or true negative rate measures the proportion of negatives which are correctly identified. The PPV describes the probability of having the condition given a positive screening test result in the analyzed population. The NPV describes the probability of not having the condition given a negative screening test result in the analyzed population.

## Results

### Profile Development

#### Gene expression profiles by histological subtype

Whole genome expression arrays were performed in tumor samples of patients of the training cohort, and expression profiles of SCC and adenocarcinoma tumor types were compared one gene at a time using the one-sample *t*-test. After single-step Bonferroni adjustment, 727 genes were identified to be differentially expressed by more than two fold in either histological subtype relative to the reference pool (Tables A, B, C and D in Table S1). Of these 727 genes, five were up-regulated and 195 down-regulated in patients with adenocarcinoma, and 13 were up-regulated and 516 down-regulated in patients with SCC.

**Figure 1:**
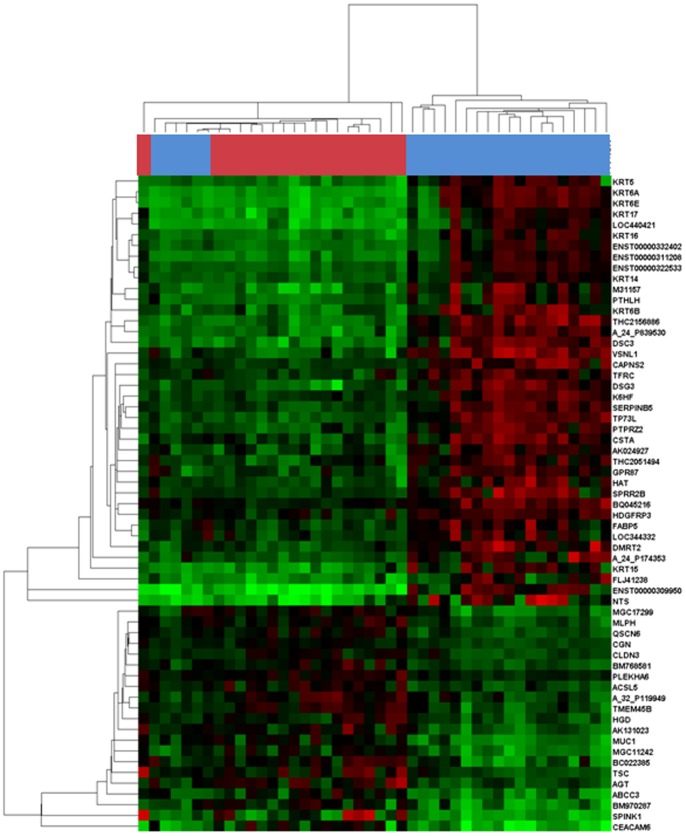
Distinct transcriptional profiles of SCC and adenocarcinoma histological subtypes. The Prediction Analysis of Microarray algorithm identified 61 genes that defined a molecular signature for each histological subtype. The modulators’ dendrogram represents an unsupervised hierarchical clustering analysis of 19 adenocarcinoma and 25 SCC based on their gene expression profile. The heat map was color coded using red for up-regulation and green for down-regulation from a lung cancer reference pool. On the *top of the heap map,* colours correspond to gene expression profiles of SCC samples (blue) versus adenocarcinoma samples (red).

Additionally, a second independent evaluation of mRNA differential expression was performed by discriminant microarray data analysis to minimize false-positive findings. The Prediction Analysis of Microarray (PAM) algorithm identified 61 genes that defined a molecular signature able to discriminate adenocarcinoma from SCC samples (figure 1). Of these 61 genes, 56 matched deregulated genes found by the previously performed one-sample t-test, and were therefore selected for further analysis and validation.

#### MicroRNA expression profile by histological subtype

MicroRNAs TLDA arrays were performed in tumor samples of patients of the training cohort. Nine miRNAs (miR-149, miR-205, miR-375, miR-378, miR-422a, miR-483-5p, miR-494, miR-601 and miR-708) were found to be differentially expressed between the SCC and adenocarcinoma histological subtypes by a FDR-corrected threshold of 0.05. Eight of these 9 miRNAs were over-expressed in SCC compared to adenocarcinoma, and one (miR-375) was over-expressed in adenocarcinoma compared to SCC (Table 3).

**Table 3:** Identification of down-regulated mRNA from deregulated miRNAs in SCC and adenocarcinoma.

	Target genes predicted	Prediction programs
***Over-expressed miRNAs in SCC***		
***miR-149***	*ABCC3*	miRanda, miRWalk, TargetScan
	*MUC1*	TargetScan
	*CEACAM6*	miRanda, miRWalk, TargetScan
	*CGN*	miRanda
	*CLDN3*	miRanda
***miR-205***	*ACSL5*	miRanda, TargetScan
	*MLPH*	miRWalk, TargetScan
	*CEACAM6*	miRanda, miRWalk, TargetScan
***miR-378***	*TMEM45B*	miRanda
***miR-422a***	*TMEM45B*	miRanda
***miR-483-5p***	*TMEM45B*	miRanda, TargetScan
***miR-494***	*ACSL5*	TargetScan
	*MLPH*	miRanda
	*CEACAM6*	miRanda
***miR-601***	*MLPH*	miRanda, miRWalk, TargetScan
***miR-708***	*CEACAM6*	miRanda, TargetScan
***Over-expressed miRNAs in adenocarcinoma***		
***miR-375***	*DSC3*	miRanda, miRWalk, TargetScan
	*KRT6A*	miRanda
	*DMRT2*	miRanda, miRWalk

#### MicroRNA target prediction

**Figure 2:**
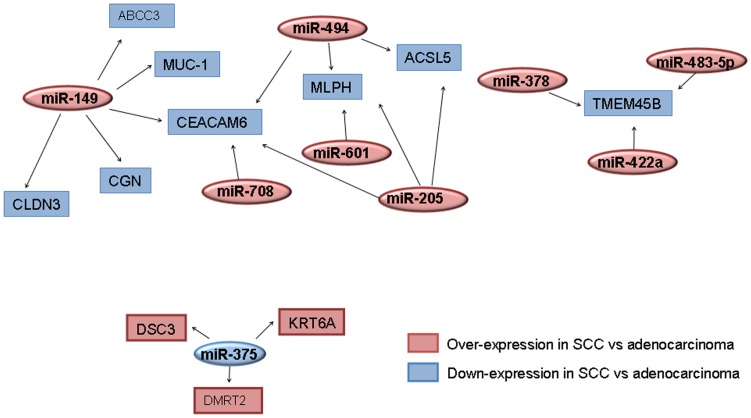
MicroRNA-mRNA target networks. The graph shows the transcriptional targets of differently expressed miRNAs in SCC and adenocarcinoma tumor types using three web databases of miRNA target prediction (miRANDA, TargetScan and miRWalk). The default colour scheme used to represent expression level is red/blue (red for over-expression of mRNAs or miRNAs in SCC versus adenocarcinoma and blue for down-expression of mRNAs or miRNAs in SCC versus adenocarcinoma). The *arrows* indicate mRNA repression by the *connected miRNAs*. Squares represent deregulated mRNAs and ovals represent differentially expressed miRNAs in lung adenocarcinoma and SCC.

Eleven of the 56 genes (20%) found to be deregulated by tumor type in our study were found to be putative targets of at least one of the 9 miRNAs also identified to be differentially expressed in our study population according to histological subtype (SCC versus adenocarcinoma). For the 8 over-expressed miRNAs in SCC, 8 mRNA (*CEACAM6, CGN, CLDN3, ABCC3, MLPH, ACSL5, TMEM45B* and *MUC1*) were predicted as targets by several algorithms. These genes were found to be down-regulated in SCC compared to adenocarcinoma in our study (Table 2). Three of these 8 genes (*CEACAM6, MLPH* and *TMEM45B)* were predicted targets of more than one of these miRNAs (figure 2). As shown in table 2, three genes (*DSC3, KRT6A* and *DMRT2*) were predicted as targets of miR-375, the miRNA upregulated in adenocarcinoma, and these genes were consistently down-regulated in this histological subtype.

### Profile Validation

#### Validation of gene differential expression by quantitative RT-PCR

Eleven deregulated genes (*CEACAM6, CGN, CLDN3, ABCC3, MLPH, ACSL5, TMEM45B, MUC1, DSC3, DMRT2* and *KRT6A),* identified as putative target genes of deregulated miRNAs in our study, were then selected for further validation by rtPCR both in the training cohort and in an independent cohort of patients.

**Figure 3:**
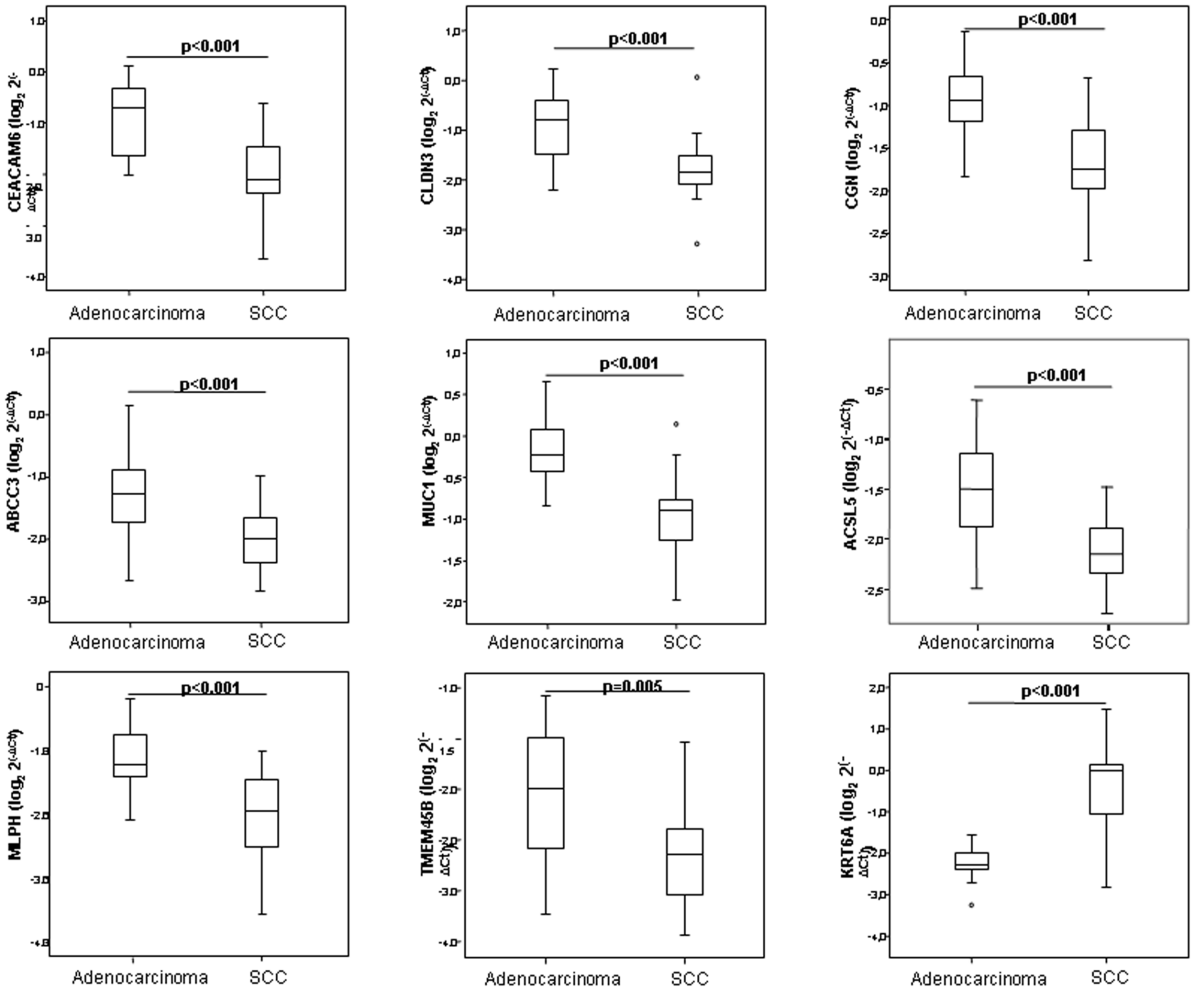
Experimental validation of deregulated mRNA in the training cohort. To validate genes identified as differentially expressed by tumor histology in the microarray data, relative expression levels of mRNAs were quantified by real-time PCR using the ΔCt method by *B2M* as housekeeping gene. The plots show median ΔCt values of validated genes in patients with adenocarcinoma versus SCC. Data derived from RT-qPCR are presented as log_2_ 2^−ΔCt^ values. *P* value below 0.05 was considered significant.

In the training cohort, 9 of the 11 genes tested were confirmed to be differentially expressed by histological subtype by quantitative PCR (figure 3). *CEACAM5*, *CLDN3, CGN, ABCC3, MUC1, ACSL5*, *MLPH* and *TMEM45B* were significantly down-regulated in SCC, and *KRT6A* was significantly down-regulated in adenocarcinoma.

**Figure 4:**
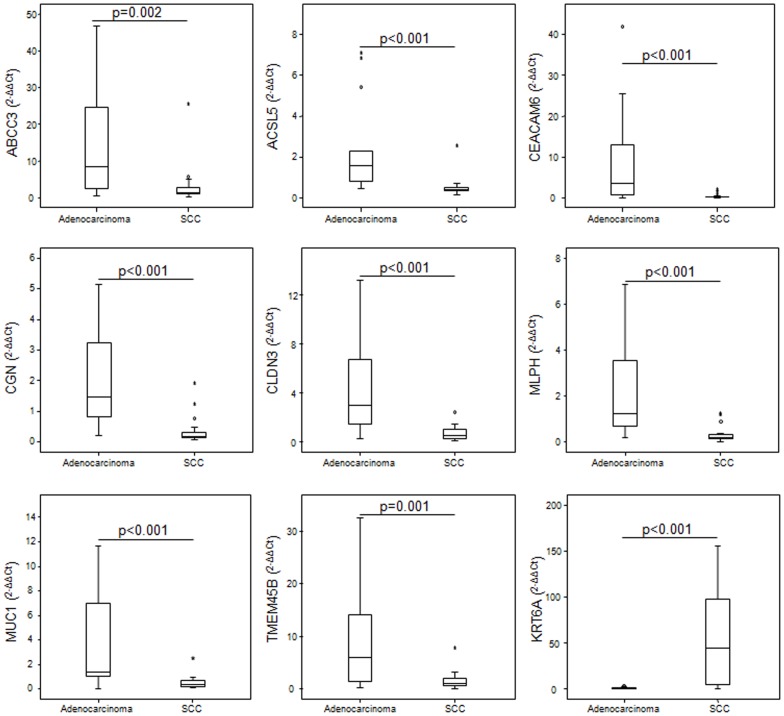
Validation of deregulated mRNA in an independent cohort. Expression of nine mRNAs was validated by real-time PCR in an independent cohort of NSCLC patients. mRNA expression levels were determined in tumor samples and paired normal lung tissue from lung cancer patients and relative expression by histological subtype was assessed. Median ΔΔCt values were determined in the validated genes in patients with adenocarcinoma and SCC. Data derived from RT-qPCR are presented as 2^−ΔΔCt^ values. *P* value below 0.05 was considered significant.

**Figure 5:**
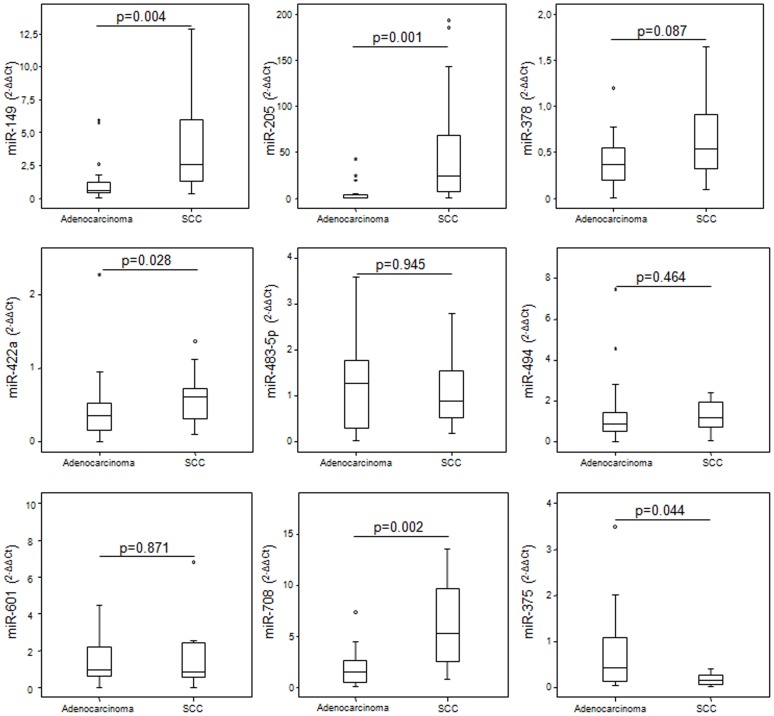
Relative quantification of deregulated microRNAs in the independent validation cohort. Expression of deregulated miRNAs was evaluated in the validation cohort. MicroRNA expression levels were determined in tumor and paired normal lung tissue of lung cancer patients and relative expression by histological subtype was assessed. Median ΔΔCt values were determined in nine miRNAs in patients with adenocarcinoma versus SCC. Data derived from RT-qPCR are presented as 2^−ΔΔCt^ values. *P* value below 0.05 was considered significant.

Based on results obtained in the training cohort, 9 mRNA and 9 miRNAs were selected for further validation by Taqman-based RT-qPCR in tumor and matched normal tissue from an independent cohort of lung cancer patients. In this cohort, expression patterns of mRNAs were consistent with those quantified in the training cohort. Expression patterns by histological subtype of all 9 mRNAs tested resembled those observed in the training cohort, and differences observed among subgroups were all statistically significant (figure 4). Regarding the 9 miRNAs, five (miR-149, miR-205, miR-378, miR-422a and miR-708) were found to be significantly over-expressed in SCC and miR-375 was significantly over-expressed in adenocarcinoma (figure 5).

#### Correlation between miRNA and mRNA expression

**Figure 6:**
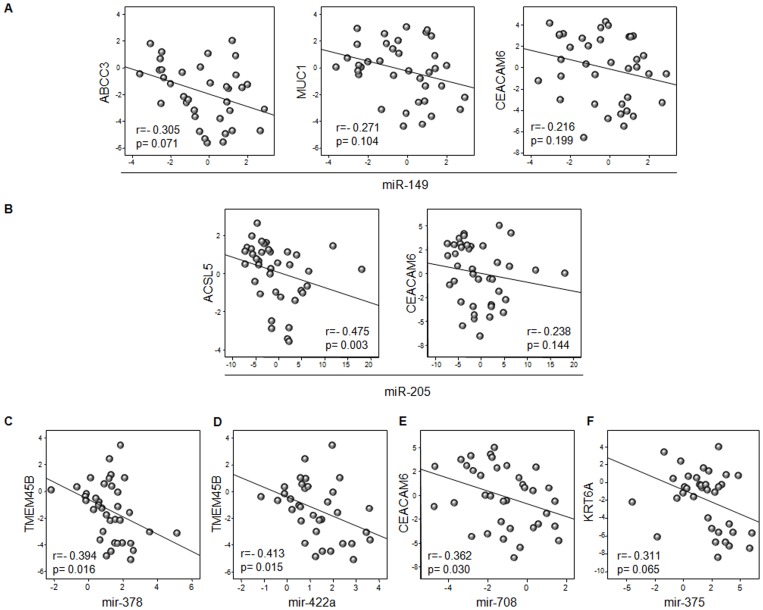
Spearman’s correlation between miRNA and target gene expression in patients with lung adenocarcinoma or squamous cell carcinoma. Expression of the 6 validated miRNAs and that of their putative target genes was measured in each patient in the validation cohort. The significance of the inverse association between each of these miRNA/mRNA couples was assessed by the Spearman’s correlation coefficient. P values less than 0.05 were considered statistically significant. A) Relationships between *ABCC3*, *MUC1* and *CEACAM6* with miR-149. B) Relationships between *ACSL5* and *CEACAM6* with miR-205. C) Relationship between *TMEM45B* and miR-378. D) Relationship between *TMEM45B* and miR-422a. E) Relationship between *CEACAM6* and miR-7018. F) Relationship between *KRT6A* and miR-miR-175.

**Figure 7:**
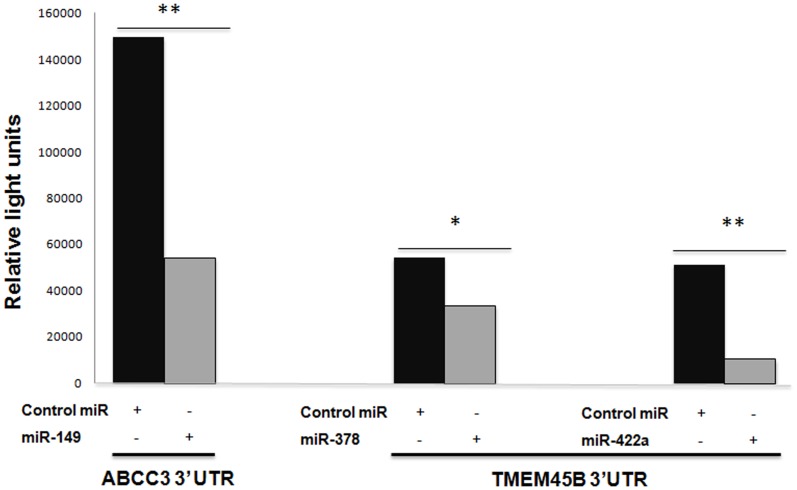
3′-UTR reporter assay for miR target validation. HEK 293 cells were transfected with luciferase reporter vector containing the 3′ UTR region of ABCC3 and TMEM45B. Reporter vectors were co-transfected with a miRN mimic or control miRN mimic. Following 24 h incubation, luciferase activity was measured. *p<0.05 and **p<0.001 by *t*-test.

To study the functional relevance of miRNAs in the regulation of specific mRNAs identified as potential biomarkers, we analyzed in the validation cohort the correlation between miRNAs and predicted target-mRNAs expression in each patient (figure 6). An inverse correlation was observed between *ABCC3, MUC1* and *CEACAM6* and miR-149 expression levels. Moreover, higher levels of *CEACAM6* were associated with lower levels of miR-205 and miR-708, being the correlation significant for miR-708 (r = −0362; p = 0.030). *ACSL5* and *KRT6A* had a statistically significant correlation with miR-205 (r = −0.475; p = 0.003) and miR-375 (r = −0.311; p = 0.065), respectively. *In the case of* TMEM45*B, significant correlations were found for miR-378 and miR-422a (r = *−*0.394, p = 0.016 and r = *−*0.413, p = 0.015, respectively).* These results suggest a potential role of miRNAs in the regulation of these genes. Subsequently, some of these targets were tested using luciferase reporter gene assays. We achieved that overexpression of miR-149 in HEK 293 cells down regulates the luciferase activity of reporter construct containing the ABCC3 3-UTR (figure 7). This shows that miR-149 binds directly to this target RNA and inhibits their expression. In addition, overexpression of miR-378 and miR-422a significantly inhibit TMEM45B expression (figure 7).

### Diagnostic Performance of Selected Genes to Discriminate SCC from Adenocarcinoma Histological NSCLC Subtypes

**Figure 8:**
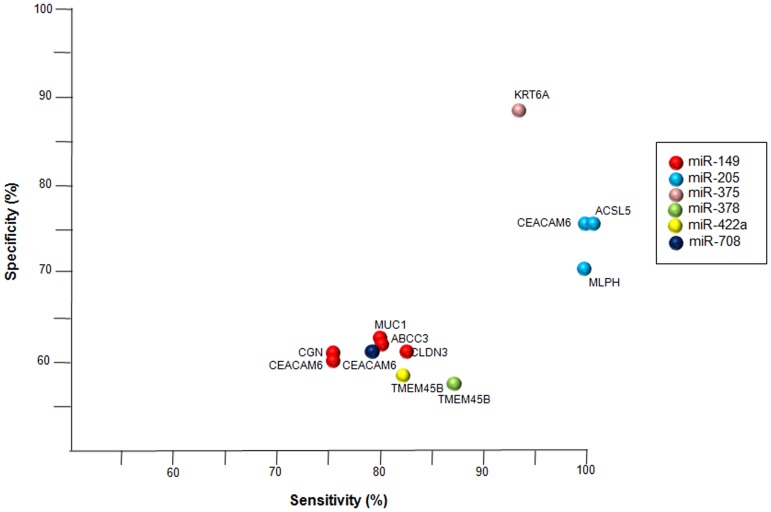
Sensitivity and Specificity of miRNA-mRNA target networks. Plot showing specificity and sensitivity of validated miRNAs in combination with predicted mRNAs to discriminate between SCC and adenocarcinoma. The colours represent down-regulated mRNA from six deregulated miRNAs in SCC or adenocarcinoma.

Finally, we evaluated the specificity and sensitivity of these six validated miRNAs in combination with their predicted mRNAs to discriminate between SCC and adenocarcinoma (figure 8). The best performance was observed for *KRT6A,* as a target of miR-375, with sensitivity and specificity values of 94.1% and 88.9%, respectively. Good diagnostic performance was also observed for *CEACAM6, ACSL5 y MLPH,* as targets of miR-205, with lower specificity values (71.4–76.2%) but higher sensitivity (100%). Finally, *TMEMB45B,* as target of miR-378, showed a sensitivity of 87.5% and a specificity of 57.7%. The other miRNA/mRNA couples revealed lower sensitivity and specificity values, although they were greater than 75% and 50% in all cases, respectively (Table S2).

## Discussion

In this study we analyzed the mRNA and miRNA expression signatures of patients with different subtypes of NSCLC. This allowed us to construct a robust transcriptional profile of lung adenocarcinoma and SCC. Our results not only indicate the existence of a mRNA and/or miRNA expression patterns that are able to distinguish between SCC and adenocarcinoma, but also that the altered gene expression signature is partly caused by specific miRNA deregulation.

First, we analyzed by two approaches the whole genome expression microarray data to minimize false positives. We examined differential gene expression levels by discriminate microarray data analysis and by the one-sample *t*-test. Fifty-six genes were found to be significantly deregulated by both analyses and were therefore selected for further evaluation and validation. Remarkably, several of them had been previously implicated in relevant biological processes (according to gene ontology) in lung cancer. For example, some genes of the *KRT* family, which were down-regulated in adenocarcinoma, are involved in several critical cell functions such as cell migration, growth and proliferation [21]. Second, miRNA profiling identified 9 miRNAs that were differentially expressed among the two histological subtypes of NSCLC studied. Six of them (miR-149, miR-205, miR-375, miR-378, miR-422a and miR-708) were further validated in an independent cohort of NSCLC patients as biomarkers able to discriminate adenocarcinoma and SCC. To assess whether these miRNAs could be directly regulating some of the 56 deregulated genes identified, several broadly used algorithms were used. Eleven of these 56 genes (20%) were thus predicted to be putative targets of at least one of the six miRNAs found to be differentially expressed in SCC compared to adenocarcinoma. Moreover, some of the predicted target genes were regulated by more than one of these miRNAs. Finally, nine (82%) of the 11 target genes were further validated by qRT-PCR in the initial training set of 44 patients and subsequently in an independent validation cohort. Other mechanisms (i.e. epigenetic regulation) are likely involved in the regulation of the remaining differentially expressed genes.

Functional studies indicate that miRNAs participate in the regulation of almost every cellular process investigated and that changes in their expression are observed in diseases such as cancer. Bioinformatic predictions indicate that mammalian miRNAs can regulate approximately 30% of all protein-coding genes [22]. Thus, post-transcriptional regulation by microRNA may be involved in the development of the different histological phenotypes of NSCLC, and these miRNA/mRNA couples identified may prove to be useful tools for diagnostic purposes or as potential novel targets for lung cancer therapy. Consistent with this, 2 of the 6 miRNAs we validated in our study (miR-205 and miR-708) had been proposed by other investigators, in combination with miR-210, as biomarkers to improve the early detection of SCC [23], [24]. In addition, Huang *et al.* found recently that miR-375 along with miR-29a accurately discriminate squamous cell lung cancer from NSCLC [25].

To date, several protein profiles have been proposed for classifying NSCLC, including TTF1, napsin A, p63 and KRT5/6 or TRIM29, CEACAM5, SLC/A5, MUC1 and KRT5/6, among others [26], [27]. In our work, mRNA expression levels of *CEACAM6, CGN, CLDN3, ABCC3, MLPH, ACSL5, TMEM45B and MUC1,* were significantly lower in SCC compared to adenocarcinoma. Some of these genes are involved in cell-cell adhesion which is a critical process in the formation and maintenance of tissue patterns during development, and also a critical process during invasion and metastasis, one of the hallmarks of cancer [28], [29], [30], [31].

CEACAM6 is an intercellular adhesion molecule that is over-expressed in a wide variety of human tumours and represents an important determinant of cancer progression [32], [33], [34], [35]. Over-expression of CEACAM6 has been reported to affect cell migration, cell invasion, and cell adhesion *in vitro,* and agents blocking CEACAM6 decreased the number of migrating cells in preclinical models [28]. Furthermore, over-expression of CEACAM6 is associated with a poorer prognosis of patients with colorectal adenocarcinoma following surgical resection, and is a very useful marker for the follow-up of these patients in the clinic [36]. Duxbury *et al*. have demonstrated that CEACAM6 plays a significant role in anoikis resistance [37]. Regarding lung cancer, and consistent with our observations, other investigators have reported higher *CEACAM6* expression levels in adenocarcinoma as compared to SCC tumours [34]. Moreover, *CEACAM6* is a putative target for 3 of the miRNAs found to be upregulated in SCC in our study (miR-149, miR-205 and miR-708).

On the other hand, we have showed that adenocarcinoma phenotype is associated with a higher expression of *CLD3* and *CGN*, whereas SCC often loses the expression of these genes. These genes are not likely regulated by miRNAs. CLDN3 and CGN are involved in tight junctions, which are a hallmark of polarized epithelial cells, providing a barrier to control the diffusion of integral membrane proteins from apical to basolateral membrane surfaces [29], [30], [31].

ABCC3 is a member of the superfamily of ATP-binding cassette (ABC) transporters. Several ABC transporters are linked to lung cancer, such as ABCC1, ABCC3, ABCA3 and ABCC5 [38]. ABCC3 is a member of the MRP subfamily which is involved in multi-drug resistance to chemotherapeutic agents, playing a major role in the failure of cancer therapy [39]. O’Brien *et al*. identified that ABCC3 amplification correlates with lack of clinical benefit from taxane-containing regimens in HER2-amplified breast cancer [40]. In addition, a small set of nine gene-signatures, in which ABCC3 is included, has been recently proposed for sub-classification of NSCLC [41]. In our study, we found that *ABCC3* expression was higher in adenocarcinoma than in SCC and found that the expression of this gene may be regulated by miRNA-149. Therefore, the *ABCC3* gene could serve as a predictive biomarker of response to chemotherapy in this subtype of NSCLC. Nonetheless, these results need to be validated in larger prospective cohorts to adequately address their clinical application.

Mucine-1, MUC1, is a transmembrane glycoprotein that is normally expressed on the apical surface of mammary epithelial cells. Nevertheless, its aberrant expression has been observed in patients with tumours of glandular epithelial origin, as breast, ovary, lung, and prostate cancers, among others [42]. Therefore, this protein could be a potential target for therapeutic interventions in cancer. Moreover, the detection of MUC1 has been linked to the simultaneous expression of multiple angiogenic factors (as VEGF) and with an aggressive tumour behaviour [43], [44], [45], [46]. In addition, there is convincing evidence that this oncoprotein confers resistance to genotoxic anticancer agents [47]. On the other hand, it is interesting to note that MUC1 in combination with other proteins have been proposed as immunohistochemical tests for subclassification of lung adenocarcinoma and SCC [26]. Here, we found also higher expression levels of MUC1 in lung adenocarcinoma than in SCC tumours. In addition, we observed that the low levels of transcripts in SCC was associated with higher expression levels of miR-149.

At the present time, the precise functions of ACSL5, MLPH and TMEM45B in cancer remain unknown. ACSL5 is a member of the ACS family, which converts fatty acid to acyl-CoA. This protein is highly expressed in uterus and spleen, and in trace amounts in normal brain, but has markedly increased levels in malignant gliomas [48]. Moreover, it has been described that ACSL5 plays a dominant role *in vitro* in the biosynthesis of mitochondrial cardiolipin and could be involved in cancer cell survival [49]. In the case of MLPH, this protein is involved in the transport of melanosomes [50]. Overexpression of MLPH has been observed in epithelial-enriched tissues in mice, such as kidney, lung, skin, small intestine, and stomach [50]. MLPH is the only trafficking protein known to be regulated by aldosterone at transcriptional level. In our study, we observed that these genes were up-regulated in adenocarcinoma versus SCC. *ACSL5* may be regulated by miR-205. However, we could not confirm a significant association between *MLPH* and miR-205 expression. On the other hand, *TMEM45B* expression levels were negatively correlated with expression levels of miR-378 and miR-422a.

In our work, the inverse relationship between miR-375 and *KRT6A* expression levels was consistently observed across histologies. The expression of this gene was significantly increased in SCC versus adenocarcinoma. Consistent with this finding, *KRT6A* is related with the maintenance of the epidermal integrity. Indeed, KRT6A is a member of the keratin protein family and is related to the epidermalization of squamous epithelium [51].

In conclusion, we have identified and validated miRNA-dependent mRNA distinct profiles able to discriminate between SCC and adenocarcinoma histological subtypes of NSCLC. The present results contributes to the progress of our understanding of the molecular pathogenesis of lung cancer and may provide important evidence to improve classification of poorly differentiated NSCLC, as well as potential novel biomarkers for personalized treatment strategies.

## Supporting Information

Table S1 Differentially expressed genes in SCC and adenocarcinoma relative to the reference pool. T-test was performed with false discovery rate (FDR) control estimated using the single-step Bonferroni procedure. Genes that passed the t-test filter were subjected to a second filter. Only genes showing a 2-fold change were selected as differentially expressed.
(XLSX)

Table S2 The specificity and sensitivity of validated miRNAs in combination with their predicted mRNAs to discriminate between SCC and adenocarcinoma. Sensitivity measures the proportion of actual positives which are correctly identified and specificity measures the proportion of negatives which are correctly identified. The PPV describes the probability of having the condition given a positive screening test result in the analyzed population. The NPV describes the probability of not having the condition given a negative screening test result in the analyzed population.
(DOC)
