# Supplementary material for: MicroRNA-Dependent Regulation of Transcription in Non-Small Cell Lung Cancer
Source: PLoS One. 2014 Mar 13;9(3):e90524. doi: 10.1371/journal.pone.0090524 (PMC3953115; doi:10.1371/journal.pone.0090524)
Supplement: Table S2 — The specificity and sensitivity of validated miRNAs in combination with their predicted mRNAs to discriminate between SCC and adenocarcinoma. Sensitivity measures the proportion of actual positives which are correctly identified and specificity measures the proportion of negatives which are correctly identified. The PPV describes the probability of having the condition given a positive screening test result in the analyzed population. The NPV describes the probability of not having the condition given a negative screening test result in the analyzed population. (DOC) [file pone.0090524.s002.doc]

Table S2

| **MicroRNA** | **Gene** | **Sensitivity (%)** | **Specificity (%)** | **PPV (%)** | **NPV (%)** |
| --- | --- | --- | --- | --- | --- |
| ***Over-expression of miRNAs and down-regulating target genes in SCC*** | | | | | |
| ***miR-149*** | ABCC3 | 80.0 (IC 95% 58.4-91.9) | 62.5 (IC 95% 42.7-78.8) | 64.0 (IC 95% 44.5-79.8) | 78.9 (IC 95% 56.7-91.5) |
|  | MUC1 | 80.0 (IC 95% 58.4-91.9) | 62.5 (IC 95% 42.7-78.8) | 64.0 (IC 95% 44.5-79.8) | 78.9 (IC 95% 56.7-91.5) |
|  | CEACAM6 | 76.2 (IC 95% 54.9-89.4) | 60.9 (IC 95% 40.8-77.8) | 64.0 (IC 95% 44.5-79.8) | 73.7 (IC 95% 51.2-88.2) |
|  | CGN | 76.2 (IC 95% 54.9-89.4) | 60.9 (IC 95% 40.8-77.8) | 64.0 (IC 95% 44.5-79.8) | 73.7 (IC 95% 51.2-88.2) |
|  | CLDN3 | 83.3 (IC 95% 60.8-94.2) | 61.5 (IC 95% 42.5-77.6) | 60.0 (IC 95% 40.7-76.6) | 84.2 (IC 95% 62.4-94.5) |
| ***miR-205*** | ACSL5 | 100 (IC 95% 54.9-89.4) | 76.2 (IC 95% 54.9-89.4) | 79.2 (IC 95% 59.5-90.8) | 100 (IC 95% 80.6-100) |
|  | MLPH | 100 (IC 95% 82.4-100) | 71.4 (IC 95% 50.0-86.2) | 75.0 (IC 95% 55.1-88.0) | 100 (IC 95% 79.6-100) |
|  | CEACAM6 | 100 (IC 95% 83.2-100) | 76.2 (IC 95% 54.9-89.4) | 79.2 (IC 95% 59.5-90.8) | 100 (IC 95% 80.6-100) |
| ***miR-378*** | TMEM45B | 87.5 (IC 95% 64.0-96.5) | 57.7 (IC 95% 38.9-74.5) | 56.0 (IC 95% 37.1-73.3) | 88.2 (IC 95% 65.7-96.7) |
| ***miR-422a*** | TMEM45B | 82.4 (IC 95% 59.0-93.8) | 59.3 (IC 95% 40.7-75.5) | 56.0 (IC 95% 37.1-73.3) | 84.2 (IC 95% 62.4-94.5) |
| ***miR-708*** | CEACAM6 | 80.0 (IC 95% 58.4-91.9) | 62.5 (IC 95% 42.7-78.8) | 64.0 (IC 95% 44.5-79.8) | 78.9 (IC 95% 56.7-91.5) |
| ***Over-expression of miRNAs and down-regulating target genes in adenocarcinoma*** | | | | | |
| ***miR-375*** | KRT6A | 94.1 (IC 95% 73.0-99.0) | 88.9 (IC 95% 71.9-96.1) | 84.2 (IC 95% 62.4-94.5) | 96.0 (IC 95% 80.5-99.3) |

PPV: Positive predictive value; NPV: Negative predictive value.
